# Correlation of transient elastography with hepatic venous pressure gradient in patients with cirrhotic portal hypertension: A study of 326 patients from India

**DOI:** 10.3748/wjg.v23.i4.687

**Published:** 2017-01-28

**Authors:** Ashish Kumar, Noor Muhammad Khan, Shrihari Anil Anikhindi, Praveen Sharma, Naresh Bansal, Vikas Singla, Anil Arora

**Affiliations:** Ashish Kumar, Noor Muhammad Khan, Shrihari Anil Anikhindi, Praveen Sharma, Naresh Bansal, Vikas Singla, Anil Arora, Institute of Liver, Gastroenterology, and Pancreatico-Biliary Sciences, Ganga Ram Institute for Postgraduate Medical Education and Research, Sir Ganga Ram Hospital, New Delhi 110060, India

**Keywords:** Portal hypertension, Cirrhosis, Clinically significant portal hypertension, Liver stiffness, Transient elastography, FibroScan

## Abstract

**AIM:**

To study the diagnostic accuracy of transient elastography (TE) for detecting clinically significant portal hypertension (CSPH) in Indian patients with cirrhotic portal hypertension.

**METHODS:**

This retrospective study was conducted at the Institute of Liver, Gastroenterology, and Pancreatico-Biliary Sciences, Sir Ganga Ram Hospital, New Delhi, on consecutive patients with cirrhosis greater than 15 years of age who underwent hepatic venous pressure gradient (HVPG) and TE from July 2011 to May 2016. Correlation between HVPG and TE was analyzed using the Spearman’s correlation test. Receiver operating characteristic (ROC) curves were prepared for determining the utility of TE in predicting various stages of portal hypertension. The best cut-off value of TE for the diagnosis of CSPH was obtained using the Youden index.

**RESULTS:**

The study included 326 patients [median age 52 (range 16-90) years; 81% males]. The most common etiology of cirrhosis was cryptogenic (45%) followed by alcohol (34%). The median HVPG was 16.0 (range 1.5 to 30.5) mmHg. Eighty-five percent of patients had CSPH. A significant positive correlation was noted between TE and HVPG (rho 0.361, *P* < 0.001). The area under ROC curve for TE in predicting CSPH was 0.740 (95%CI: 0.662-0.818) (*P* < 0.01). A cut-off value of TE of 21.6 kPa best predicted CSPH with a positive predictive value (PPV) of 93%.

**CONCLUSION:**

TE has a fair positive correlation with HVPG; thus, TE can be used as a non-invasive modality to assess the degree of portal hypertension. A cut-off TE value of 21.6 kPa identifies CSPH with a PPV of 93%.

**Core tip:** Clinically significant portal hypertension (CSPH), which is defined as hepatic venous pressure gradient (HVPG) ≥ 10 mmHg, causes major complications of cirrhosis. HVPG is invasive, so a non-invasive tool to diagnose CSPH is needed. This study of 326 Indian patients tested the diagnostic accuracy of transient elastography (TE) for detecting CSPH. We observed a significant positive correlation between TE and HVPG (rho 0.361, *P* < 0.001). The area under the receiver operating characteristic curve for TE in predicting CSPH was 0.740. A cut-off value of TE of 21.6 kPa best predicted CSPH with a positive predictive value of 93%. Thus, TE can be used as a non-invasive modality to assess the degree of portal hypertension.

## INTRODUCTION

The end result of ongoing injury to the liver due to any cause is hepatic fibrosis, leading to architectural changes and often cirrhosis[1]. Progressive hepatic fibrosis is the most important factor leading to parenchymal dysfunction and the development of portal hypertension. The measurement of portal hypertension is important, as a progressive increase in portal pressure is believed to predict various complications of cirrhosis[2-4]. Hepatic venous pressure gradient (HVPG) is the ideal method for the measurement of portal hypertension and the prediction of complications[5]. Porto-systemic collaterals develop at HVPG greater than 10 mmHg[6], and variceal bleeding could occur from varices when the pressure increases to greater than 12 mmHg[7]. An HVPG greater than 10 mmHg is used as the cut-off for “clinically significant portal hypertension” (CSPH)[8]. However, HVPG is an invasive procedure that requires care and training.

Many non-invasive direct and indirect tests have been reported that are able to predict the presence of CSPH in patients with cirrhosis with considerable accuracy. The ideal non-invasive diagnostic test for portal hypertension should be simple, inexpensive, widely accessible and reliable in measurement and interpretation and provide clinically reliable and relevant information about the degree of portal hypertension. Transient elastography (TE) is a novel, non-invasive, ultrasound-based technology that allows the measurement of liver stiffness. Established evidence indicates that TE has good sensitivity and specificity for diagnosing liver fibrosis and cirrhosis and has been popular over the past few years[9,10]. Recently, many European studies have reported a fairly good correlation between liver stiffness and portal hypertension, suggesting that it could be a good non-invasive tool for evaluation of portal hypertension[11]. However, none of the studies are from South Asia where the etiological profile of cirrhosis is different from other regions of the world.

In the present study, we aimed to identify a possible correlation between TE and HVPG in Indian cirrhosis patients and to investigate whether TE can serve as a non-invasive diagnostic test to identify patients who have CSPH with a reliable cut-off TE value.

## MATERIALS AND METHODS

### Patients

The study was conducted at the Institute of Liver, Gastroenterology, and Pancreatico-Biliary Sciences, Sir Ganga Ram Hospital, New Delhi, on patients who underwent HVPG and TE from July 2011 to May 2016. The study conformed to the Helsinki declaration of 1975 as revised in 1983. The study was retrospective on prospectively enrolled patients during this period.

**Inclusion criteria:** Consecutive patients with cirrhosis greater than 15 years of age who underwent HVPG and TE were included in the study. Both these procedures should have been performed within an interval of one week.

**Exclusion criteria:** The following patients were excluded from the study: (1) patients with non-cirrhotic cause of portal hypertension; (2) patients with acute-on-chronic liver failure; (3) patients with an invalid reading of TE or whose HVPG was not possible due to technical reasons; (4) patients who received beta blocker therapy in the past two weeks; and (5) concomitant extrahepatic malignancy.

### Evaluation

Each included patient underwent a detailed evaluation in terms of demographic parameters, etiology of cirrhosis, assessment of severity of liver disease (CTP, MELD), and assessment of severity of portal hypertension. A standard methodology was followed for the measurement of liver stiffness by TE and measurement of HVPG.

### Liver stiffness measurement by TE

Liver stiffness measurement was performed using a FIBROSCAN^®^ (Echosens, Paris, France) device in accordance with the manufacturer’s recommendations. Measurements were performed on the right lobe of the liver through intercostal spaces with the patient lying in a supine position with the right arm in maximal abduction. The tip of the transducer probe was covered with coupling gel and placed on the skin between the rib bones at the level of the right lobe of the liver. When the target area was located, the operator pressed the probe button to commence the measurements. The measurement depth was between 25 and 65 mm. Ten successful measurements were performed on each patient. The results were expressed in kilopascals (kPa). The median value was considered as the liver stiffness. Interquartile range/median < 30% and success rate > 60% were considered as good quality criteria for TE. Patients with significant ascites underwent large volume paracentesis before liver stiffness measurement. All liver stiffness measurements were performed by a single operator.

### Hepatic venous pressure gradient

HVPG was measured by introducing a 7-French Swan-Ganz catheter *via* the transfemoral or transjugular approach into a major hepatic vein as previously described[12]. The catheter was advanced until it was wedged into the hepatic vein. The occluded position of the catheter was assessed by the absence of reflux after the injection of 2 mL of contrast medium and the appearance of a sinusoidogram. A mean of three HVPG readings was obtained. If there was a difference of greater than 1 mm Hg between the readings, all the recordings were discarded, and fresh readings were obtained.

### Statistical analysis

Continuous variables are expressed as the median with ranges, and discrete variables are expressed as numbers (%). Correlations between variables were analyzed using Spearman’s correlation test. Comparisons of continuous variables between two groups were performed by Mann-Whitney *U* test, and comparisons between multiple groups were performed by Kruskal-Wallis test. Fisher’s exact test or χ^2^ test was used to compare categorical variables. Receiver operating characteristic (ROC) curves were prepared to determine the utility of TE in predicting various stages of portal hypertension. The best cut-off value of TE for the diagnosis of CSPH was obtained by using the Youden index. SPSS 17 (Chicago, IL, United States) software was used for analysis.

## RESULTS

### Patients

From January 2014 to June 2016, three hundred and seventy-nine patients were screened for enrolment in the study. Of these, 326 patients were included in the study, and the remaining 53 were excluded due to following reasons: (1) patients with non-cirrhotic cause of portal hypertension (*n* = 16); (2) patients with acute-on-chronic liver failure (*n* = 27); (3) patients with invalid TE reading or whose HVPG was not possible due to technical reasons (*n* = 5); (4) patients who received beta blocker therapy in the past two weeks (*n* = 3); and (5) concomitant extrahepatic malignancy (*n* = 2).

Table 1 presents the demographic profile of patients studied. The median age was 52 years (range 16-90 years), and the majority (81%) were males. The most common etiology of cirrhosis was cryptogenic (45%) followed by alcohol (34%). Ascites was present in 51% of patients. Sixty-four percent of patients were non-bleeders, whereas the remaining had bled from varices in the past. The median CTP score was 7 (range 5-12), and the median MELD score was 12 (range 6-37). The median liver stiffness was 36 kPa with a range of 3 to 75 kPa.

**Table 1:** Demographic profile of the study population

**Parameter**	**Value (*n* = 326)**
Gender	
Males	263 (81)
Females	63 (19)
Age, yr	52 (16-90)
BMI, kg/m^2^	23 (17-41)
Etiology	
NASH/cryptogenic	148 (45)
Alcohol	110 (34)
Viral (HBV/HCV)	48 (15)
Others (including mixed etiology)	20 (6)
Ascites	
None	161 (49)
Mild	135 (42)
Moderate to tense	30 (9)
Bleeding status	
Bleeder	118 (36)
Non-bleeder	208 (64)
Hemoglobin, g/dL	10.3 (4.5-17.0)
Platelets, × 10^3^/cumm	90 (13-422)
Bilirubin, mg/dL	1.6 (0.2-11.2)
AST, IU/dL	53 (16-209)
INR	1.3 (0.9-3.2)
Serum albumin, g/dL	3.0 (1.2-4.6)
CTP score	7 (5-12)
MELD score	12 (6-37)
Varices present	293 (90)
Esophageal varices	280 (86)
Small varices	170/280 (61)
Large varices	110/280 (39)
Gastric varices	79 (24)
Small varices	52/79 (66)
Large varices	27/79 (34)
HVPG, mmHg	16.0 (1.5-30.5)
Transient elastography, kPa	36 (3-75)

All values are expressed as the median (range) or *n* (%). NASH: Non-alcoholic steatohepatitis; HBV: Hepatitis B virus; HCV: Hepatitis C virus; AST: Aspartate aminotransferase; INR: International normalized ratio; CTP: Child-Turcotte-Pugh; MELD: Model for end-stage liver disease; HVPG: Hepatic venous pressure gradient.

### HVPG

The median HVPG of all patients was 16.0 (range 1.5 to 30.5) mmHg. Table 2 shows patients categorized according to HVPG stages. Four percent patients had normal HVPG (≤ 5 mmHg), while the remaining 96% had portal hypertension (HVPG > 5 mmHg). Eighty-five percent of patients had clinically significant portal hypertension (HVPG ≥ 10 mmHg). Seventy-six percent patients had HVPG greater than 12 mmHg (severe portal hypertension, SPH), which is the threshold for variceal bleeding. In addition, 18% patients had very high HVPG (> 20 mmHg, very severe portal hypertension, VSPH).

**Table 2:** Groups according to hepatic venous pressure gradient

**HVPG (mmHg)**	***n* (%)**	**Portal hypertension (> 5 mmHg)**	**CSPH (**≥ **10 mmHg)**	**SPH (> 12 mmHg)**	**VSPH (> 20 mmHg)**
≤ 5	14 (4)	No (14, 4%)	No (48, 15%)	No (78, 24%)	No (266, 82%)
> 5 to < 10	34 (10)	Yes (312, 96%)			
≥ 10 to 12	30 (9)		Yes (278, 85%)		
> 12 to ≤ 20	188 (58)			Yes (248, 76%)	
> 20	60 (18)				Yes (60, 18%)

CSPH: Clinically significant portal hypertension; SPH: Severe portal hypertension; VSPH: Very severe portal hypertension.

### Correlation of TE with HVPG

**Figure 1:**
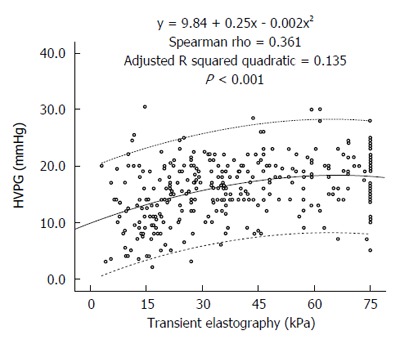
Scatterplot of transient elastography and hepatic venous pressure gradient values. HVPG: Hepatic venous pressure gradient.

A significant positive correlation was noted between liver stiffness and HVPG levels (Spearman’s rho 0.361, *P* < 0.001). Figure 1 presents the scatterplot of TE and HVPG values. The HVPG value could be predicted by the following formula:

HVPG= 9.84 + 0.25 × *TE* - 0.002 × *TE*^2^. The adjusted R squared value was 0.135.

### TE values in patients with various stages of portal hypertension

**Figure 2:**
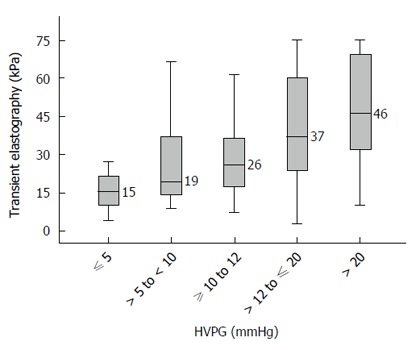
Median transient elastography values in patients with various stages of portal hypertension. HVPG: Hepatic venous pressure gradient.

Figure 2 presents the median TE values in various stages of portal hypertension. Cirrhotic patients with no portal hypertension (HVPG ≤ 5 mmHg) had a median TE value of 15.4 (range 4.1 to 75.0) kPa. Patients with HVPG > 5 to < 10 mmHg (sub-clinical portal hypertension, SCPH) had a median TE value of 19.4 (range 8.8 to 74.0) kPa. Patients with HVPG ≥ 10 to 12 mmHg had a median TE value of 25.8 (range 7.3 to 75.0) kPa. Patients with HVPG > 12 to ≤ 20 mmHg had a median TE value of 37.1 (range 2.95 to 75.0) kPa. Patients with HVPG > 20 mmHg had median TE value of 46.4 (range 10.1 to 75.0) kPa. Although these TE values were significantly different across the groups (*P* < 0.05), there was considerable overlap in the interquartile ranges between various groups (Figure 2).

### Performance of TE in predicting various stages of portal hypertension

**Figure 3:**
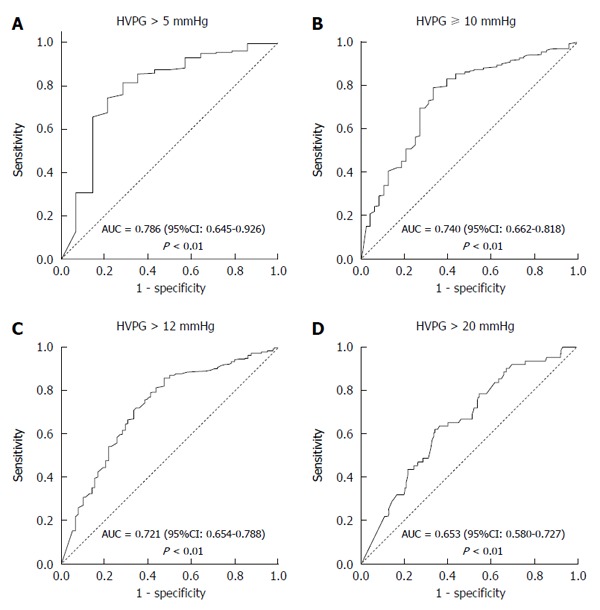
Receiver operating characteristic curves of transient elastography for predicting various stages of portal hypertension. HVPG: Hepatic venous pressure gradient.

Figure 3 presents the ROC curves of TE for predicting the various stages of portal hypertension. Ninety-six percent of patients had portal hypertension (HVPG > 5 mmHg). TE proved to be an excellent non-invasive modality in predicting portal hypertension with an area under the ROC curve of 0.786 (95%CI: 0.645-0.926) and a *P-*value < 0.01 (Figure 3A). Eighty-five percent patients had clinically significant portal hypertension (HVPG ≥ 10 mmHg). The area under the ROC curve for TE in predicting CSPH was 0.740 (95%CI: 0.662-0.818) (*P* < 0.01) (Figure 3B). Seventy-six percent patients had HVPG > 12 mmHg, which is the threshold for variceal bleeding. The area under the ROC curve for TE in predicting HVPG > 12 mmHg was 0.721 (95%CI: 0.654-0.788) (*P* < 0.01) (Figure 3C). Eighteen percent of patients had very high portal hypertension (HVPG > 20 mmHg). The area under the ROC curve for TE in predicting HVPG > 20 mmHg was 0.653 (95%CI: 0.580-0.727) (*P* < 0.01) (Figure 3D).

### Cut-off TE value for predicting CSPH

**Figure 4:**
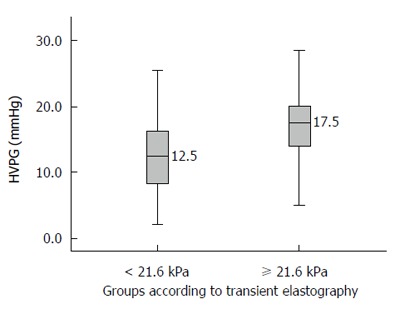
Median hepatic venous pressure gradient values in patients with transient elastography values less than and greater than 21.6 kPa. HVPG: Hepatic venous pressure gradient.

When HVPG is ≥ 10 mmHg, it is known as clinically significant portal hypertension (CSPH). Most complications of portal hypertension, such as varices, ascites, encephalopathy, and bleeding, occur at or above this value. The area under the ROC curve for TE to diagnose CSPH was 0.740 (95%CI: 0.662-0.818). A cut-off value of TE of 21.6 kPa was obtained by using Youden index to best predict CSPH. The sensitivity, specificity, positive predictive value, negative predictive value, and accuracy of a TE value ≥ 21.6 to diagnose CSPH were 79%, 67%, 93%, 35%, and 77%, respectively (Table 3). The median HVPG in patients with a TE values ≥ 21.6 was 17.5 mmHg (Figure 4).

**Table 3:** Predictive values of transient elastography for the prediction of clinically significant portal hypertension (HVPG ≥ 10 mmHg)

**TE cut-off value (mmHg)**	**CSPH (*n*)**	**No CSPH (*n*)**	**Total (*n*)**	**Sensitivity (95%CI)**	**Specificity (95%CI)**	**PPV (95%CI)**	**NPV (95%CI)**	**Accuracy (95%CI)**	**LR+ (95%CI)**	**LR-(95%CI)**
≥ 21.6	219	16	235	79% (74%-83%)	67% (52%-80%)	93% (89%-96%)	35% (25%-46%)	77% (72%-82%)	2.4 (1.6-3.5)	0.3 (0.2-0.4)
< 21.6	59	32	91							
Total	278	48	326							

TE: Transient elastography; CSPH: Clinically significant portal hypertension; PPV: Positive predictive value; NPV: Negative predictive value; LR+: Likelihood ratio positive; LR-: Likelihood ratio negative.

## DISCUSSION

In the present study, we showed that in patients with cirrhosis, TE has a fair positive correlation with HVPG, and TE can thus be used as a non-invasive modality to assess the degree of portal hypertension. The TE values increase progressively as portal pressure increases from normal through SCPH, CSPH, SPH and VSPH. We also found that a cut-off TE value of 21.6 kPa has 93% positive predictive value in diagnosing CSPH.

Numerous previous studies have correlated TE with HVPG (Table 4). In these studies, the AUROC curve for prediction of CSPH varied between 0.78 and 0.99. The optimal cut-offs ranged between 13.6 and 65.3 kPa with PPV typically greater than 80%. However, most of these studies used a small number of patients, and almost all of these studies were performed in Europe with none from South Asia where the etiological profile of cirrhosis is different from the West. The largest of these studies was by Reiberger et al[13] from Austria, who retrospectively correlated TE and HVPG in 502 patients. They identified a very high correlation of TE with HVPG (*r* = 0.799; *P* < 0.0001). Compared with their study, our correlation coefficient was lower (*r* = 0.361). Two possible reasons could explain this difference in the strength of correlation between their study and ours. The first reason is the difference in the etiological profiles of patients in the two studies. Their study had more patients with a viral etiology (56%) that typically exhibits a better correlation, whereas our study had more patients with a NASH/cryptogenic and alcohol etiology, which typically has poorer correlation. The second possible reason for the difference in the strength of correlation between their study and ours was that their patients had less severe liver disease compared with our patients. The mean HVPG of their cohort was 12.6 (± 7.6) mmHg, whereas the median HVPG in our patients was 16.0 mmHg. Their median liver stiffness value was 26.4 kPa, whereas that in our study was 36 kPa. TE and HVPG correlate better when liver disease is less advanced. As observed from the scatterplot of our study (Figure 1) and also from the scatterplot of Reiberger’s study[13], the slope of the trend line on the left side of the graph when the disease is less severe is steeper, indicating a better correlation compared with the right side when the trend line is flatter, indicating a poorer correlation at higher TE values. Vizzutti et al[14] also found that the correlation was excellent for HVPG values less than 10 or 12 mmHg (*r* = 0.81 and *r* = 0.91, respectively); however, the linear regression analysis was not optimal for HVPG values ≥ 10 mmHg (*r* = 0.59) or ≥ 12 mmHg (*r* = 0.37). In advanced portal hypertension, it is not only the liver fibrosis but also the extrahepatic factors, such as the increase in blood flow due to hyperdynamic circulation, that contribute to portal hypertension[15].

**Table 4:** Various studies of the diagnostic performance of transient elastography for clinically significant portal hypertension

**Ref**.	**Place**	**Year**	**Number of patients**	**Correlation coefficient (r) or r^2^ of TE with HVPG**	**AUROC (95%CI)**	**Best cut-off of TE for CSPH**
Carrión et al[33]	Spain	2006	124	0.840	0.94	-
Vizzutti et al[14]	Italy	2007	61	0.810	0.99 (0.92-0.99)	13.6 (PPV 97%)
Lemoine et al[34]	France	2008	92	0.530	0.84 (0.80-0.88)	34.9 for alcohol (PPV 98%)
						20.5 for HCV (PPV 88%)
Bureau et al[35]	France	2008	150	0.858	0.945 (0.904-0.987)	21 (PPV 92%)
Sánchez-Conde et al[36]	Spain	2011	38	0.460	0.80 (0.64-0.97)	14 (PPV 84%)
Reiberger et al[13]	Austria	2012	502	0.794	0.817 (0.752-0.891)	18 (PPV 86%)
Llop et al[20]	Spain	2012	97	0.552	0.840 (0.748-0.933)	21 (PPV 81%)
Berzigotti et al[37]	Spain	2013	117	-	0.883 (0.824-0.943)	21.1 (sensitivity 65%)
Hong et al[38]	South Korea	2013	59	0.496	0.851	21.95 (PPV 87%)
Salzl et al[28]	Austria	2014	88	0.765	0.87	16.8 (sensitivity 90%)
Augustin et al[39]	Spain	2014	40	-	-	25
Zykus et al[31]	Lithuania	2015	107	0.750	0.949	17.4 (accuracy 89%)
Procopet et al[32]	Europe	2015	202	-	0.94 (0.89-0.99)	21.1 (accuracy 90%)
	(Multicentric)					
Kitson et al[40]	Australia	2015	95	0.380	0.90 (0.83-0.97)	29.0 (PPV 100%)
Elkrief et al[25]	France	2015	79	-	0.78 (0.58-0.98)	65.3 (PPV 100%)
Schwabl et al[41]	Austria	2015	226	0.836 and 0.846	0.957 & 0.962	16.1 (accuracy 89% & 90%)
Hametner et al[29]	Austria	2016	236	-	0.92 (0.86-0.96)	24.8 (PPV 98%)
This study	India	2016	326	0.361	0.740 (0.662-0.818)	21.6 (PPV 93%)
Total			2515			Weighted mean: 21.8

TE: Transient elastography; HVPG: Hepatic venous pressure gradient; AUROC: Area under receiver operating characteristic; CSPH: Clinically significant portal hypertension; PPV: Positive predictive value.

There was an urgent need for a South Asian study on the correlation of TE - HVPG because the results of Western studies may not apply to the South Asian population where the etiological and the clinical profile of chronic liver disease differs from the West. Alcohol consumption and the prevalence of diabetes [a major risk factor for nonalcoholic fatty liver disease (NAFLD)] has been steadily increasing in South Asia[16]. The International Diabetes Federation has revised its estimates of the number of people with the diabetes in South-East Asia from 50 million in 2009 to 78.3 million in 2015, with a projection of 140 million by 2040[17,18]. Although cirrhosis mortality has been steadily decreasing globally and especially in the West, it has been steadily increasing in India since 1980. With 188,575 liver cirrhosis deaths in 2010, India ranks number one in the world in liver cirrhosis deaths, accounting for almost one-fifth (18%) of global liver cirrhosis deaths[19]. With such a huge population of liver cirrhosis patients in India whose etiology differs from the West, an easy and non-invasive modality for portal hypertension estimation is urgently needed for treating these patients. Our study attempts to address this need for Indian and South Asian populations.

Most of the complications of cirrhosis are typically related more to CSPH compared with any other factor. Although HVPG measurement is the gold standard for diagnosing CSPH, it is not in common use because it is invasive, not widely available, and expensive, thus hindering its broad use in diagnostic and therapeutic algorithms in patients with cirrhosis with CSPH. Our study found that a TE value of 21.6 kPa is a good cut-off for predicting CSPH with a PPV of 93%. Seventeen additional studies have calculated the cut-off TE values for diagnosing CSPH (Table 4). These studies were performed in patients with different disease severities and different proportions of viral cirrhosis, and their cut-off values ranged from 13.6 kPa to 65.3 kPa. The weighted mean of cut-off from all these studies was 21.9 kPa, which is very similar to what we obtained in our study. Adding our study to the pool of studies, the weighted mean for the best cut-off TE value for diagnosing CSPH is 21.8 kPa.

Notably, Llop et al[20] from Spain provided two cut-offs of TE instead of one to predict CSPH. They showed that a cut-off of 13.6 kPa was sufficiently sensitive to exclude CSPH, and a cut-off of 21 kPa was sufficiently specific to include CSPH. They suggested that values in between these limits (which were found in 35% of their patients) were not useful. Some authors believe that the use of a single TE cut-off, although simple, limits the value of TE to predict CSPH. The use of at least two cut-offs reproduces the clinical thinking in which a diagnostic test commonly provides three outputs: the condition is included, excluded or “further tests are needed”[15]. Thus, TE, using these three outputs, might be useful to select these suspicious patients with cirrhosis for HVPG measurement. However, we believe that multiple cut-offs may lead to confusion, and the single cut-off with a high PPV is best for guiding primary physicians in the community to make treatment decisions.

A recent meta-analysis[11], which included 5 studies of the diagnostic accuracy of TE for significant portal hypertension, also indicated that TE had a high accuracy for the detection of significant portal hypertension. The hierarchical summary receiver-operating characteristic (HSROC) for the diagnosis of significant portal hypertension by TE was 0.93 (95%CI: 0.90-0.95). The Fagan plot analysis showed that TE could be used to diagnose significant portal hypertension (when pre-test probability = 50%), with 81% probability of correctly diagnosing significant portal hypertension following the ‘‘positive’’ measurement. Furthermore, a ‘‘negative’’ measurement was also informative, as significant portal hypertension was present in only 11% of patients. However, when the pre-test probability of significant portal hypertension was as low as 25%, the probability of correctly identifying significant portal hypertension decreased markedly. This finding suggests that an accurate selection of patients is necessary to exploit the performance of TE at its best[11].

Other newer and promising noninvasive modalities are being developed for diagnosing portal hypertension, such as two-dimensional shear wave elastography (2D-SWE)[21-25]; acoustic radiation force impulse (ARFI)[26-28]; VITRO Score (Von Willebrand Factor Antigen/Thrombocyte Ratio)[29]; aspartate aminotransferase/platelet ratio index (APRI)[30]; spleen elsatography[23-27,31]; and serum tests, such as Fibrosis-4, and Lok score[32]. These tests have their own advantages and disadvantages. However, to date, very few studies have been performed on them for correlation with HVPG, so their routine use cannot be recommended outside of clinical trials.

There could be a few limitations in our study. First, it is a retrospective study, so the study may suffer from selection bias. We included only those patients who underwent HVPG and TE during the study period; hence, our patients may not represent the entire population of patients with cirrhosis, as most included patients have moderate to severe portal hypertension. A prospective study design, which includes all consecutive patients of cirrhosis, regardless of degree of portal hypertension, would have been a better study design and more representative of the cirrhotic population of the community. A second limitation could be the lack of follow-up. Follow-up data on complications of portal hypertension would have further validated our results of TE cut-off for CSPH.

In conclusions, our study demonstrated that in patients with cirrhosis, TE has a fair positive correlation with HVPG; thus, TE can be used as a non-invasive modality to assess the degree of portal hypertension. The TE values increase progressively as portal pressure increases from normal through SCPH, CSPH, SPH and VSPH. A cut-off TE value of 21.6 kPa has 93% positive predictive value in diagnosing CSPH. This cut-off will be very useful in diagnosing CSPH and making appropriate treatment decisions in places where HVPG is not available or when patients are unwilling to undergo HVPG due to its invasiveness. As a reliable and non-invasive procedure, TE is a promising and worthy tool to translate into routine clinical practice for detecting CSPH. TE could be integrated in the detection of CSPH in untreated patients for portal hypertension. Further large prospective studies are needed to prospectively validate the findings of our study and also to determine whether TE can be used in monitoring the hemodynamic response and the effect of drugs reducing portal pressure.

## COMMENTS

### Background

Clinically significant portal hypertension (CSPH), which is defined as hepatic venous pressure gradient (HVPG) ≥ 10 mmHg, causes major complications of cirrhosis. HVPG is invasive and not always available, so a noninvasive tool to diagnose CSPH is needed. Many studies have correlated transient elastography (TE) with HVPG, but none of them are from South Asia where the etiological profile of cirrhosis differs from other regions of the world.

### Research frontiers

TE is a novel, noninvasive, ultrasound-based technology that allows measurements of liver stiffness. Established evidence indicates that TE has good sensitivity and specificity for diagnosing liver fibrosis and cirrhosis and has been popular over the past few years. The present study tested the diagnostic accuracy of TE for detecting CSPH in Indian patients.

### Innovations and breakthroughs

The present study showed that in patients with cirrhosis, TE has a fair positive correlation with HVPG; thus, TE can be used as a non-invasive modality to assess the degree of portal hypertension. The TE values increase progressively as portal pressure increases from normal through subclinical portal hypertension (SCPH), CSPH, severe portal hypertension (SPH) and very severe portal hypertension (VSPH). In addition, a cut-off TE value of 21.6 kPa has 93% positive predictive value in diagnosing CSPH.

### Applications

This study suggests that TE, which is a reliable and non-invasive procedure, is a promising and worthy tool to translate into routine clinical practice for detecting CSPH. A TE cut-off value of 21.6 kPa is very useful in diagnosing CSPH and making appropriate treatment decisions in places where HVPG is not available or when patients are unwilling to undergo HVPG due to its invasiveness. Thus, TE could be integrated in the detection of CSPH in untreated patients of portal hypertension.

### Terminology

HVPG represents the approximate gradient between portal vein and intra-abdominal vena cava pressure. Measurement of the hepatic venous pressure gradient HVPG is currently the best available method to evaluate the presence and severity of portal hypertension. TE, known by the brandname FibroScan, is a non-invasive test to quantify liver stiffness. Liver stiffness increases with increasing liver fibrosis.

### Peer-review

The authors of this paper have demonstrated that in patients with cirrhosis, TE has a fair positive correlation with HVPG; thus, TE can be used as a non-invasive modality to assess the degree of portal hypertension. Further large prospective studies are needed to prospectively validate the findings of this study and also to determine whether TE can be used in monitoring the hemodynamic response and the effect of drugs reducing portal pressure.
